# Natural head orientation and spatial hearing with symmetric frontal maskers

**DOI:** 10.3389/fnins.2026.1724840

**Published:** 2026-05-14

**Authors:** Heesung Park, Nathan C. Higgins, Erol J. Ozmeral

**Affiliations:** Department of Communication Sciences and Disorders, University of South Florida, Tampa, FL, United States

**Keywords:** head movement, head orientation, listening engagement, listening strategy, spatial hearing, speech perception

## Abstract

The purpose of this study was to evaluate the effect of natural, undirected head orientation on speech perception in the presence of interfering speech maskers that were symmetrically arranged to minimize the better-ear advantage. We also examined the characteristics of natural head motion under these conditions. Fourteen normal-hearing adults participated in continuous number categorization tasks under both head-fixed and head-free conditions. Three parameters were measured across different spatial listening configurations: (1) speech reception threshold (SRT) in both co-located and spatially separated masker conditions (±30° azimuth), (2) accuracy with spatially separated maskers at a fixed target-to-masker ratio (TMR), and (3) functional spatial boundary (FSB) in an adaptive masker-location condition. No significant differences in speech perception performance were observed between head-fixed and head-free conditions across all listening configurations. However, performance changes relative to the head-fixed condition were significantly correlated with head-orientation magnitude in the co-located SRT and FSB conditions. Exploratory analyses further indicated that larger head rotations were sometimes associated with performance improvements, whereas smaller rotations occasionally accompanied performance decrements. These observations may reflect complex interactions between dynamic spatial cues produced by head motion and moment-to-moment variations in task engagement. However, these observations warrant cautious interpretation and may provide a basis for future investigations into the role of natural head movement in spatial listening.

## 1 Introduction

In real-world listening, listeners move their head to hear better, especially when engaged in multi-talker conversations. Apart from the benefits of visual cues provided by head orientation (e.g., lip-reading, facial expression) (Grant and Seitz, 2000; Higgins et al., 2023), head movement also enhances spatial hearing, improving sound localization and speech intelligibility in the presence of multiple interferers (Wallach, 1940; Perrett and Noble, 1997a; Grange et al., 2018). When static spatial cues are ambiguous in the horizontal and vertical planes, head movements introduce changes in both interaural disparities and spectral filtering patterns. Changes in interaural disparities can resolve front- back confusion, while time–varying spectral cues primarily support vertical localization (Kock, 1950; Blauert, 1997; Perrett and Noble, 1997b; Wightman and Kistler, 1999; Middlebrooks, 2015). While head movement transiently resets the ongoing perceptual organization of auditory streams, it may, under conditions of clear spatial separation, give rise to a more effective reorganization of streaming by providing new spatial cues (Kondo et al., 2012).

When listeners were allowed to move their head during sound localization test, Thurlow et al. (1967) found spontaneously significant propensity for yaw head rotation to aid in horizontal localization. However, subsequent studies have reported contradictory findings in horizontal localization tasks and have found no significant benefit in related spatial hearing tasks. Many studies demonstrated that head movement significantly improved the horizontal accuracy and reduced angular error (Honda et al., 2013; Gulli et al., 2022; Valzolgher et al., 2024b). On the other hand, Cooper et al. (2008) presented evidence that rapid head turns smeared spatial cues, thereby increasing localization errors. Furthermore, Lertpoompunya et al. (2024) observed no significant improvement in spatial digit detection accuracy with head movement compared to the head-fixed condition.

Regarding speech perception in noise, sequential studies by Grange and colleagues demonstrated the head orientation benefit obtained by turning one's head away from the target speech presented from the front. First, they clinically verified the benefit predicted by a model based on the additive combination of better-ear listening and binaural unmasking, indicating that spatial release from masking (SRM) varied as a function of head orientation (Jelfs et al., 2011; Grange and Culling, 2016a). In a follow-up study with normal hearing listeners, they found that directing the head 30 degrees away from the target speech location resulted in a statistically significant improvement of 3–5 dB in speech reception threshold (SRT) in the presence of a single speech-shaped noise azimuthally located within the rear hemifield. Moreover, in a simulated real-world listening environment with multiple interfering sound sources, a significant but modest improvement exceeding 1 dB was also observed (Grange and Culling, 2016b). Additionally, their findings highlighted that instructed listeners, who were informed about the potential head orientation benefits, showed improved SRT of 3.3 dB and SRM of 1.6 dB with increased head movement compared to their uninstructed condition, where listeners exhibited modest or often suboptimal head movements (Grange and Culling, 2016a; Grange et al., 2018).

Other studies, however, have not shown significant head orientation benefits for speech intelligibility. In an azimuthally diffuse noise condition excluding a target location, natural head movement showed no significant correlation with speech discrimination, subjective difficulty, or effortfulness in a normal-hearing group (Tetard et al., 2023). Gifford et al. (2018) also found no head orientation benefit for speech intelligibility at any target location (0°, +90° or −90° azimuth) in either a bilateral or bimodal cochlear implant (CI) group. Liang et al. (2022) reported no significant differences in SRTs across simulated head deviations of −30°, −60°, and −90° from a target speaker located on the right side (+90° azimuth), in both virtual automotive and quasi-free field conditions. Furthermore, Hadley et al. (2019) reported that listeners rarely adopted substantial head reorientation strategies even as noise levels increased. Similarly, Hendrikse et al. (2018) observed that head movements were minimal in the absence of visual cues and found no clear evidence that the performance benefit observed in the audiovisual condition was attributable to head motion itself.

Because there are a limited number of studies that have addressed the effect of natural head turns on speech perception in the presence of multiple competing interferers, and these studies have mixed results, there remains a gap in our understanding of the benefits associated with head rotation in complex auditory scenes. Prior studies did not directly compare head-fixed and head-free conditions, nor did they examine head orientation benefits beyond the better-ear advantage afforded by head rotation. Therefore, the present study examined the effect of natural head orientation on spatial hearing through direct comparison, rather than instructed head turns. Furthermore, we used symmetrically separated maskers in the frontal hemifield to minimize the better-ear advantage and to focus on potential release from masking driven either by optimal static spatial cues or by dynamic cues (i.e., time-varying spatial cues) arising from head movements (Kondo et al., 2014; Cho and Kidd, 2022).

To assess spatial hearing, this study included several conventional outcome measures: SRTs in co-located and spatially separated symmetric masker conditions, as well as test accuracy. We also measured spatial acuity of spatial release from masking (SRM) using the functional spatial boundary (FSB), defined as the smallest angular separation between a target and maskers required to achieve a given level of SRM (Ozmeral and Higgins, 2022). This measure is also referred to as the minimum angular separation (Peng and Sweeney, 2025). These measures were included because they represent different listening configurations and task paradigms that may differentially engage natural head-movement behavior. Accordingly, this study examined natural head orientation and movement tendencies, that is, how listeners naturally moved their heads under different listening configurations.

## 2 Materials and methods

### 2.1 Participants

Sixteen participants were initially enrolled. However, two participants were excluded as outliers because they did not fully engage with the task, which caused the adaptive procedure to frequently reach its ceiling limits across most conditions, resulting in outlier values and unreliable estimates. Fourteen total participants completed the study and were included in all data analysis (mean age = 23.1 years, range = 20–28 years; 10 females). Listeners were screened for normal hearing, with pure-tone thresholds equal to or better than 25 dB HL at octave frequencies between 0.25 and 8 kHz. The subjects reported no history of audiological or neurological pathologies, and all passed the Montreal Cognitive Assessment (MoCA; Nasreddine et al., 2005). This study protocol was approved by the Institutional Review Board (IRB) of the University of South Florida, all participants provided written informed consent prior to participation, and compensation was provided.

### 2.2 Apparatus

All tests were conducted in a double-walled sound treated booth (dimensions: 3.15 m x 3.05 m x 1.99 m) with 24 loudspeakers (Q100, KEF, England) arranged in a horizontal circular array with 15-degree spacing and a radius of 1.05 meters. Audio signals were routed from three 8-channel amplifiers (ne8250, Ashly, NY) connected to a 24-channel external sound card (MOTU 24ao, MOTU, MA). To achieve better angular resolution, a simplified version of equal power panning was applied between loudspeakers (Pulkki, 1997). Head orientation during testing was recorded using an optical tracking system (OptiTrack V120, NaturalPoint, OR) suspended from the ceiling above the central loudspeaker. This system, operating at a sampling rate of 120 Hz, tracked eight reflective markers mounted on an adjustable headband worn by the participants. All tests, measurements, and data collection were performed using custom MATLAB programming software (R2023b, The MathWorks, MA).

### 2.3 Stimuli

Continuous speech was used as the test stimulus, which was generated by randomly concatenating monosyllabic words designed for the Continuous Number Identification Test (Ozmeral et al., 2020). A male voice served as the target speech, while two different female voices acted as interfering speech. Unlike the interfering speech stream, only the target speech stream contained numerical target words (mean duration = 0.43 sec, SD = 0.04 s) ranging from 1 to 10, excluding 5 and 7. The target number presented approximately every 4.2 s. The target speech stream with a 100-ms inter-word interval was always routed from the front (0° azimuth), with a fixed presentation level of 52 dBA.

Masker streams were symmetrically arranged within a range of ±90° azimuth to minimize better-ear hearing due to the head shadow effect (Marrone et al., 2008; Oh et al., 2022). Their presentation level varied across experimental conditions, and no inter-word interval was imposed. In addition, multi-lingual background babble was presented at 47 dBA (5 dB SNR) from four loudspeakers positioned behind the listener at ±135° and ±165°. Each loudspeaker played a unique two-talker conversation in a different foreign language. These served to limit potential glimpsing cues and reduce variability in outcomes (Ozmeral and Higgins, 2022).

### 2.4 Procedure

Participants with the head-mounted optical tracking band were seated in a height-adjustable chair at the center of the array. Their pinnae aligned to the center of the loudspeakers. They were asked to detect numbers and then indicate whether the number in the target speech stream was greater or less than 5 by pressing the upward or downward arrow button on a response keypad (XK-4 stick, P.I. Engineering, MI) held in their dominant hand. A response within 2.5 seconds after the onset of each target word was regarded as the corresponding response. The study consisted of four different listening conditions. The shorthand naming convention uses ‘T' for target and ‘M' for masker, followed by their azimuths in degrees. The conditions were: (1) SRT in the co-located condition, denoted T0M0(SRT); (2) SRT with maskers spatially separated at ±30° azimuth, denoted T0M30(SRT); (3) response accuracy (%) with fixed masker level and location (±30° azimuth), denoted T0M30(%); and (4) functional spatial boundaries (°) in an adaptive masker-location condition, denoted FSB(°).

The SRTs for the fixed masker-location condition were computed in dB target-to-masker ratio (TMR) using a 2-down, 1-up adaptive procedure, which estimated a 70.7% accuracy (Levitt, 1971). TMR was calculated by subtracting the combined level of the two interferers from the target level. To do this, the target level was fixed, while the masker levels were adaptively adjusted. A single block included 12 reversals, with the threshold determined by averaging the last 9 reversals. The step size was adjusted from 2 dB to 1 dB after the first three reversals. In the T0M30(%) task, where both masker level and location were fixed, the level was set to −3 dB target-to-masker ratio (TMR) relative to the co-located SRT. Participants were presented with 50 target numbers, and speech perception performance was quantified as categorization accuracy (%). In the FSB(°) task, using the adaptive procedure, the minimum angular separation (°) between the target and maskers necessary to achieve a 3-dB SRM was computed by adjusting the separation, as outlined by Ozmeral and Higgins (2022). This 3-dB SRM was observed when maskers of a different gender from the target were positioned symmetrically around ±45° from the front (Oh et al., 2022). Setting the SRM criterion at 3 dB could provide a spatial boundary that can be further modulated by natural head orientation. Accordingly, the fixed level was set to −3 dB TMR, relative to the SRT measured in the co-located and head-fixed condition. The initial separation was 45°, and initial step size was 5°, which then narrowed to 2° after the first three reversals, following the same adaptive procedure as the SRT measure.

Following a practice session, these tasks proceeded in the sequential order of T0M0(SRT), FSB(°), T0M30(%), and T0M30(SRT). Each task included two different head behavior conditions: head-fixed and head-free. In the head-fixed condition, participants were instructed to face forward toward the front loudspeaker during the test, while in the head-free condition, they were free to move their heads naturally. The experimenter gave the following instruction: ‘You may move your head as freely as you like.' The head behavior order was counterbalanced. Moreover, each condition consisted of three trials (i.e., three repetitions of the same listening task). Therefore, the total number of tests was 24 (4 listening configurations x 2 head conditions x 3 trials).

### 2.5 Data analysis

**Figure 1:**
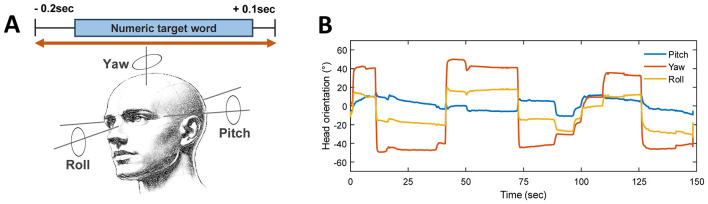
Measurement of head orientation. **(A)** Schematic illustration of the duration of numerical target words and corresponding head-rotation measurement. Orange lines illustrate the epoching window for head orientation analysis, extending from 0.2 s before target word onset to 0.1 s after target word offset. **(B)** Example of time series from a single trial of a participant illustrating head orientation changes in pitch (blue), yaw (orange), and roll (yellow). Positive angles in pitch, yaw, and roll indicate head lifting, turning to the right, and tilting to the right, respectively. Negative angles represent the opposite orientations.

Head orientation in pitch, yaw, and roll was tracked throughout the test, yielding those rotation angles around the lateral, vertical, and longitudinal axes (Figure 1A). The reference position was defined as 0° in pitch, yaw, and roll, corresponding to the head facing forward toward the front loudspeaker. The recorded data were screened for tracking artifacts, identified as abrupt simultaneous spikes across channels or samples exceeding ±70°, as the optical tracking system showed systematic underestimation at larger eccentricities and maximum observed values of approximately ±65° under our experimental configuration. Artifactual samples were replaced by interpolation, and the cleaned signals were then low-pass filtered at 3 Hz to reduce high-frequency tracking noise and jitter. Post-processing isolated head orientation data corresponding only to the numeric target period by computing the root mean square (RMS) within a time window spanning −0.2 s before target onset to +0.1 s after the target offset (Figure 1A). The head orientation RMS values were averaged across repeated trials to obtain an individual head orientation RMS, representing the magnitude of orientation independent of rotation direction. In addition, head orientation variability was quantified as the standard deviation (SD) of head orientations across target presentations, reflecting the extent of movement variability. Furthermore, to investigate temporal changes in head orientation, mean RMS and SD values were compared across the first, middle, and last eight target presentations.

A two-way repeated measures ANOVA was conducted to compare SRTs based on head orientation and masker location, followed by *post-hoc* pairwise comparisons using Bonferroni adjustment. A paired *t*-test was conducted to compare the accuracy and FSB between the head-fixed and head-free conditions. A Mann-Whitney U test was performed to compare head orientation angles between correct and incorrect responses. In addition to the analyses based on averaging the three repeated runs within each head condition, complementary trial-level analyses were performed separately for each behavioral outcome using linear mixed-effects models (LMMs). Each model included head condition (head-fixed vs. head-free) as a fixed effect and listener as a random intercept. Pearson correlation analysis was used to assess relationships among performance differences between head-fixed and head-free conditions across test conditions. Based on individual trial data, Pearson correlation analysis was conducted to assess the relationship between changes in behavioral hearing outcomes and head orientation relative to the head-fixed condition. The effect of head movement on performance, relative to the fixed condition (Δbehavioral), was computed by subtracting the mean head-fixed value (averaged across three trials) from each participant's head-free score. In parallel, the Δhead orientation RMS was defined as the difference between the head-free orientation RMS and the mean head-fixed orientation RMS.

A one-way repeated measures ANOVA and Friedman test were conducted to compare head orientation RMS across the four test conditions. A total of 42 head-free samples (14 participants × 3 trials) for each condition were divided into two subsets based on the Δbehavioral hearing outcomes: positive outcome trials, in which performance improved compared to the individual's mean head-fixed value, and negative outcome trials, in which performance declined. A linear mixed model was implemented, with trial outcome group (positive vs. negative, based on Δbehavioral), target-presentation phase (initial, middle, and final), and their interaction included as fixed effects. Target-presentation phase was treated as a repeated within-subject factor at the participant level, and within-subject correlations were modeled using an unstructured covariance matrix. Most statistical analyses were performed in IBM SPSS Statistics, version 30.0 (IBM Corp., Armonk, NY, USA), with MATLAB used for complementary analyses.

## 3 Results

### 3.1 T0M0(SRT): SRT with the co-located target and maskers at the front

**Figure 2:**
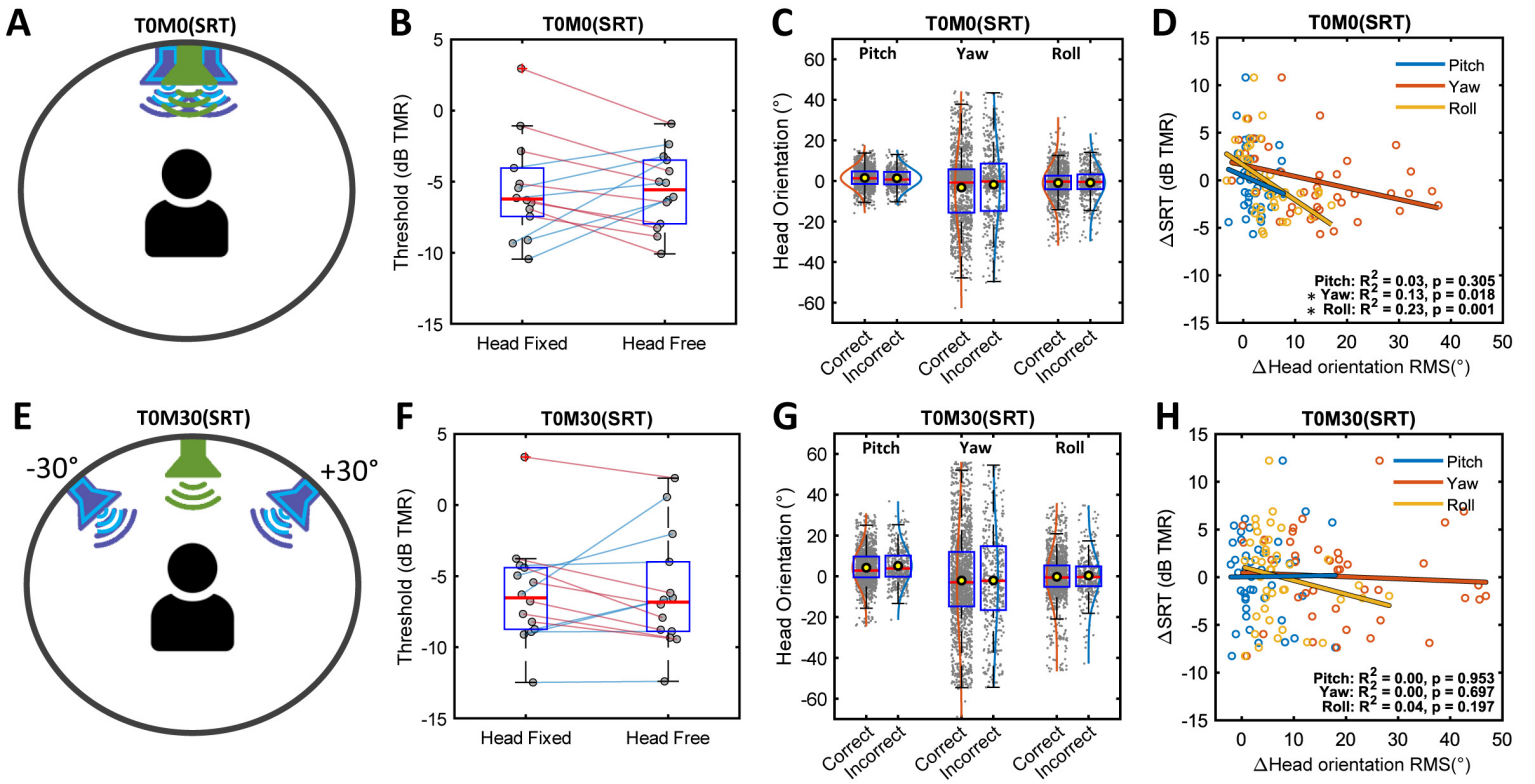
Speech reception thresholds (SRTs) in the co-located condition at the front, T0M0(SRT), and in the spatially separated condition with maskers at ±30° azimuth, T0M30(SRT). **(A, E)** Schematic illustration of the listening configurations for T0M0(SRT) and T0M30(SRT). The green loudspeaker represents the target speaker, the blue loudspeakers with light blue outlines indicate the maskers with adaptive levels, and the gray loudspeakers denote background babble noise. **(B, F)** Boxplots of SRT under head-fixed and head-free conditions. Gray scatter dots denote mean data points for each participant. Red lines represent data showing benefits from natural head movement, while blue lines indicate the disadvantages. **(C, G)** Boxplots of head orientation (pitch, yaw, and roll) for all trial responses under the head-free condition, comparing correct and incorrect responses. Yellow dots in the boxplots represent mean values, and gray dots indicate individual responses. Probability density function (PDF) curves of fitted normal distributions approximated the data distribution. **(D, H)** Relationships between Δbehavioral(SRTs) and Δhead orientation RMS in pitch, yaw, and roll. Scatter plots display all data points, with fitted regression lines for each orientation angle: blue for pitch, orange for yaw, and yellow for roll. An asterisk indicates a statistically significant correlation.

In the T0M0 condition, mean SRTs were −5.56 dB TMR for the head-fixed condition and −5.60 dB TMR for the head-free condition, showing no significant difference between the two conditions (*t*(13) = 0.046, *p* = 0.964, Bonferroni-corrected; Figure 2B). Consistent with the averaged analysis, a complementary trial-level analysis also showed no significant main effect of head condition (*F*(1, 69) = 0.004, *p* = 0.947). When all head-free responses were grouped into correct and incorrect responses, each head orientation appeared symmetrically distributed around the forward-facing pivot, despite not satisfying the normality assumption. A Mann-Whitney U test revealed no significant differences in pitch (*U* = 298,254, *p* = 0.311), yaw (*U* = 294,669, *p* = 0.165), and roll angles (*U* = 306,722, *p* = 0.898) between the two response-types (Figure 2C). However, significant negative correlations were observed between Δbehavioral(SRTs) and Δhead orientation RMS in yaw (*r*^2^ = 0.13, *p* = 0.018) and roll (*r*^2^ = 0.23, *p* = 0.001) whereas no significant correlation was found with pitch (*r*^2^ = 0.03, *p* = 0.305; Figure 2D). The negative correlation suggests that increased yaw and roll head orientations might be associated with lower (i.e., better) SRT compared to the head-fixed SRT.

### 3.2 T0M30(SRT): SRT with maskers spatially separated at ±30° azimuth

In the T0M30 condition, mean SRTs were −6.26 dB TMR for the head-fixed and −6.19 dB TMR for the head-free conditions, with no significant difference observed between the two conditions (*t*(13) = −0.094, *p* = 0.927, Bonferroni-corrected; Figure 2F). Consistent with the averaged analysis, a complementary trial-level analysis also showed no significant main effect of head condition (*F*(1, 69) = 0.006, *p* = 0.936). For the group analysis comparing correct and incorrect responses, a Mann-Whitney U test revealed no significant differences in pitch (*U* = 307,384, *p* = 0.311), yaw (*U* = 323,676, *p* = 0.165), and roll angles (*U* = 320,241, *p* = 0.898) between correct and incorrect responses (Figure 2G). Unlike the T0M0(SRT) condition, when maskers were separated from the target at ±30°, no significant correlations were found between Δbehavioral(SRTs) and Δhead orientation RMS (all *p* > 0.05; Figure 2H). SRM was computed as 0.70 dB for the head-fixed condition and 0.59 dB for the head-free condition, with no significant difference in SRTs between the co-located and separated masker conditions (both *p* > 0.05). Moreover, no significant differences in SRM were found between the head-fixed and head-free conditions (*t* (13) = 0.102, *p* = 0.920).

### 3.3 T0M30(%): accuracy with both masker level and location fixed at ±30° azimuth

**Figure 3:**
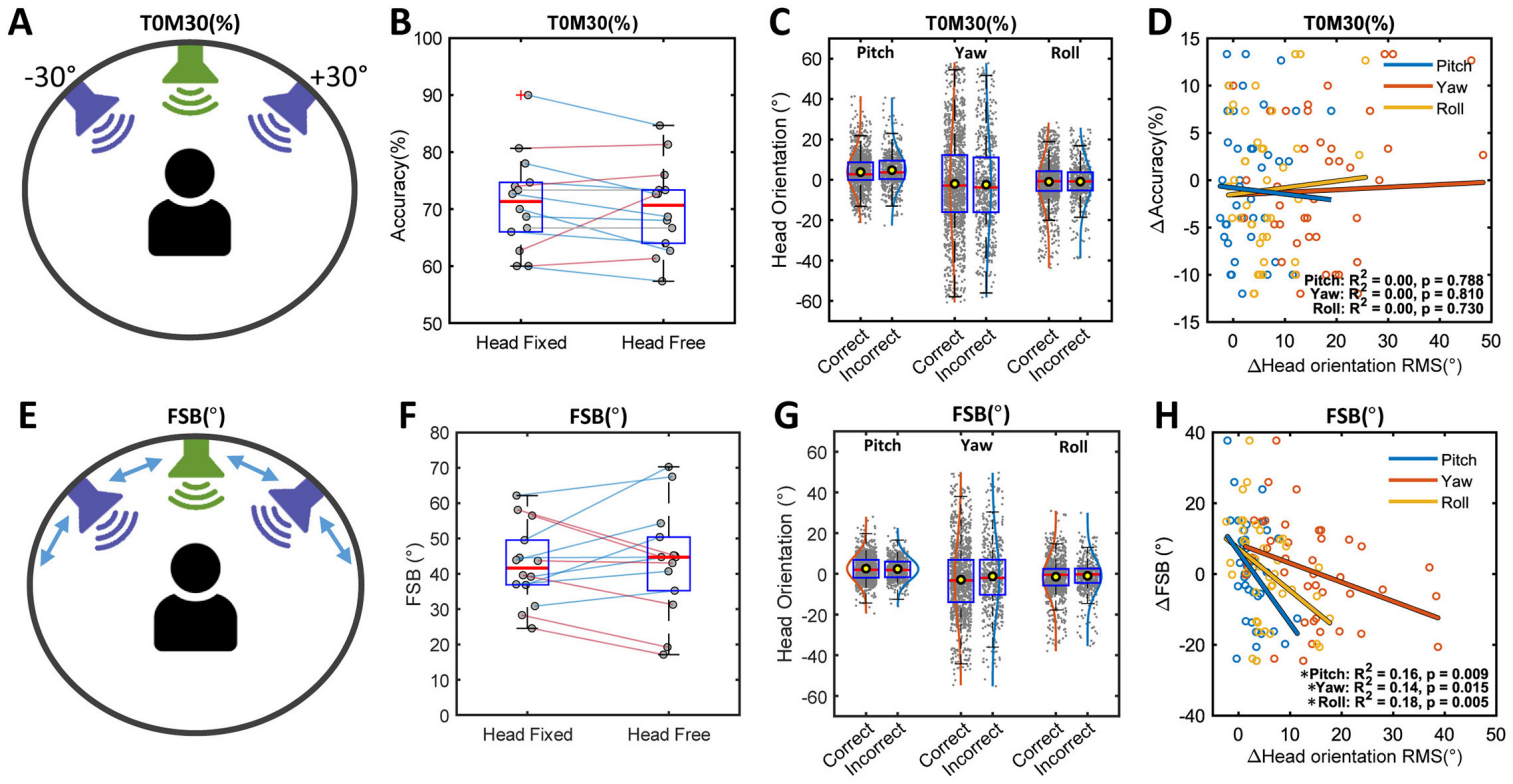
Test accuracy with both masker level and location fixed at ±30° azimuth, T0M30(%), and functional spatial boundaries with an adaptive masker-location, FSB(°). **(A, E)** Schematic illustration of the listening configurations for T0M30(%) and FSB(°). The blue loudspeakers indicate the maskers with a fixed level. In FSB(°), angular separation symmetrically decreases with correct responses but increases when participants miss. **(B, F)** Boxplots of test accuracy (%) and FSB (°), respectively, under head-fixed and head-free conditions. Red lines represent data showing benefits from natural head movement, while blue lines indicate the disadvantages. **(C, G)** Boxplots of head orientation in pitch, yaw, and roll under the head-free condition, comparing correct and incorrect responses. **(D, H)** Relationships between Δbehavioral(accuracy) and Δhead orientation RMS, and between Δbehavioral(FSB) and Δhead orientation RMS in pitch, yaw, and roll. An asterisk indicates a statistically significant correlation.

In T0M30(%), mean accuracies were 71.24% for the head-fixed and 70.19% for the head-free conditions, showing no significant difference between the two conditions (*t*(13) = 0.930, p = 0.369; Figure 3B). Consistent with the averaged analysis, a complementary trial-level analysis also showed no significant main effect of head condition (*F*(1, 69) = 0.425, *p* = 0.516). For the group analysis comparing correct and incorrect responses, a Mann-Whitney U test revealed a significant difference in pitch (*U* = 428,827, *p* = 0.010), although the magnitude of the difference was minimal at 0.96 degrees. No significant differences were observed for yaw (*U* = 451,043, *p* = 0.417) or roll angles (*U* = 459,772, *p* = 0.900; Figure 3C) and there were no significant correlations between Δbehavioral(accuracy) and Δhead orientation RMS (all *p* > 0.05; Figure 3D).

### 3.4 FSB(°): functional spatial boundaries with an adaptive masker-location

Mean FSBs were 42.5 degrees for the head-fixed and 43.5 degrees for the head-free condition, showing no significant difference between the two conditions (*t*(13) = −0.373, *p* = 0.715; Figure 3F). Consistent with the averaged analysis, a complementary trial-level analysis also showed no significant main effect of head condition (*F*(1, 69) = 0.127, *p* = 0.723). In the group analysis comparing correct and incorrect responses, a Mann-Whitney U test revealed a significant difference of approximately 1.7 degrees in yaw (*U* = 298,970, *p* = 0.019), while no significant differences were found for pitch (*U* = 317,745, *p* = 0.695) or roll (*U* = 309,570, *p* = 0.215; Figure 3G). Significant negative correlations were observed between Δbehavioral(FSB) and Δhead orientation RMS in pitch (*r*^2^ = 0.18, *p* = 0.005), yaw (*r*^2^ = 0.16, *p* = 0.009) and roll (*r*^2^ = 0.14, *p* = 0.015; Figure 3H). This demonstrates that FSB might improve with increases in head orientation.

### 3.5 Correlations of Δbehavioral across conditions

Differences in behavioral hearing outcomes between the head-fixed and head-free conditions (Δbehavioral) were computed for each test condition, and their correlations across test conditions are presented in Table 1. No significant correlations were found between any pair of conditions (all *p* > 0.05).

**Table 1:** Pearson correlations of differences in outcome measures between head-fixed and head-free conditions across test conditions (*N* = 14).

Outcome measure	Statistic	T0M0 (SRT)	T0M30 (SRT)	T0M30 (%)	FSB (°)
T0M0(SRT)	*r*	1.000	0.103	0.046	0.216
	*p*		0.726	0.875	0.457
T0M30(SRT)	*r*		1.000	−0.272	0.274
	*p*			0.347	0.343
T0M30(%)	*r*			1.000	0.252
	*p*				0.384
FSB(°)	*r*				1.000
	*p*				

### 3.6 Comparison of head orientation across listening conditions

**Figure 4:**
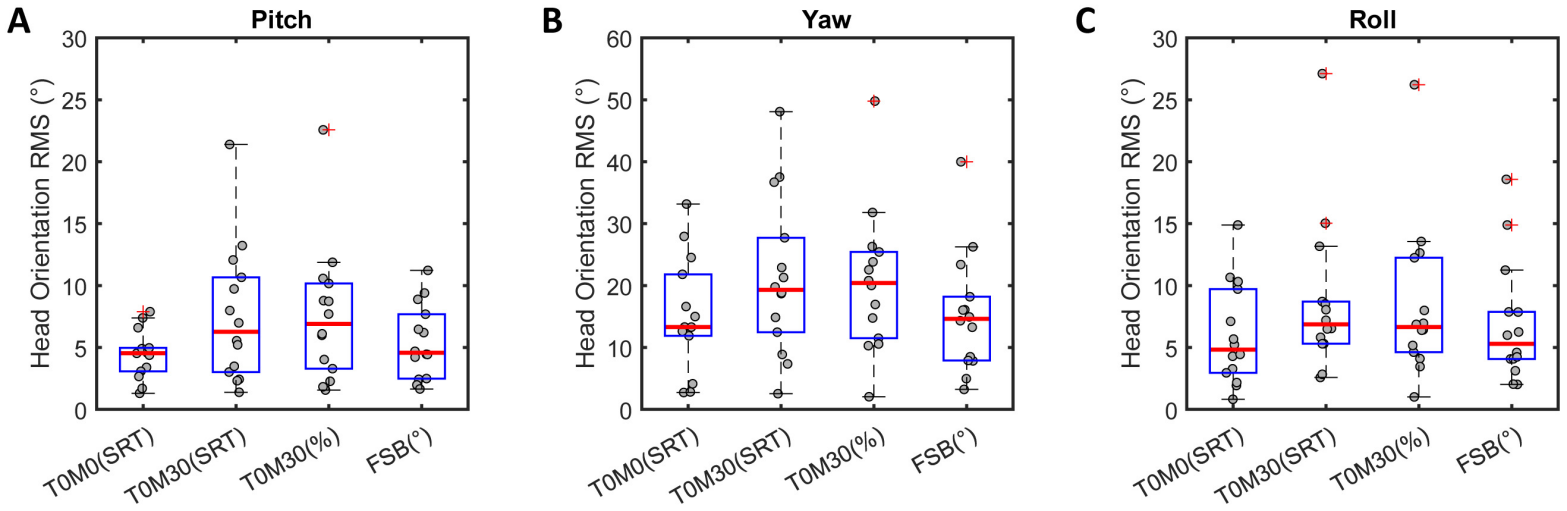
Comparison of head orientation across the four head-free conditions. Box plots display RMS head orientation for pitch **(A)** yaw **(B)** and roll **(C)** across four head-free conditions. Gray dots represent each participant's average RMS value.

A repeated-measures ANOVA revealed a significant main effect of test condition on yaw head orientation (*F*(3, 39) = 5.326, *p* = 0.004; Figure 4B) and roll orientation (*F*(3, 39) = 4.688, *p* = 0.021, Greenhouse-Geisser corrected; Figure 4C). However, *post-hoc* pairwise comparisons showed no significant differences in yaw and roll head orientation RMS (*p* > 0.05). Furthermore, a Friedman test revealed a significant main effect of test condition on pitch head orientation RMS (χ^2^(3) = 9.257, *p* = 0.026; Figure 4A), although *post-hoc* pairwise comparisons were not significant. The grand average yaw orientation RMS was significantly greater than both pitch and roll orientation RMS values in this study (both *p* < 0.001).

### 3.7 Δbehavioral-based trials comparison I: behavioral hearing outcomes

**Figure 5:**
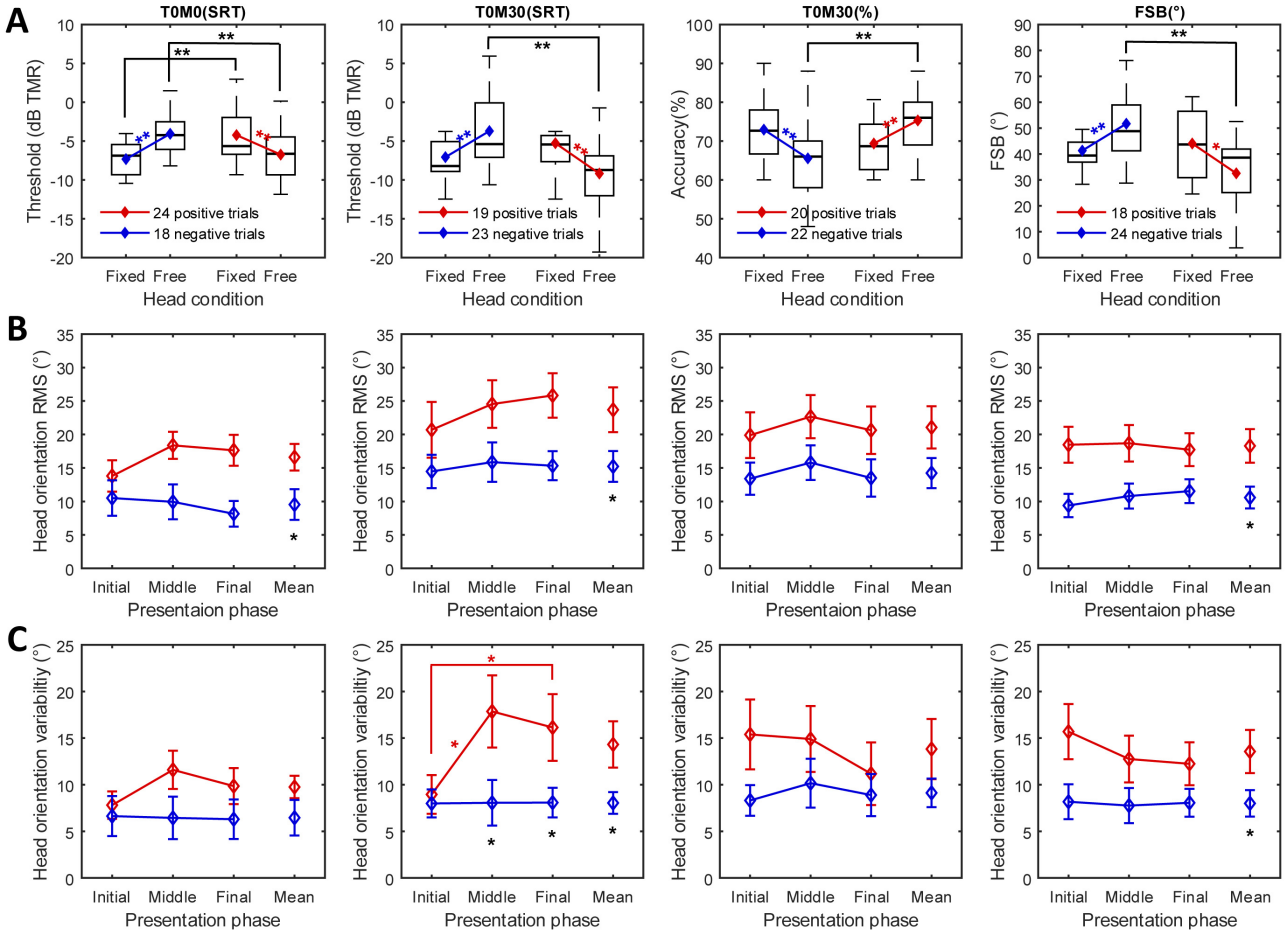
**(A)** Boxplots of behavioral hearing outcomes under head-fixed and head-free conditions, separately for positive and negative trials. The red and blue lines represent positive and negative trials, respectively, where performance in the head-free condition improved or declined relative to the head-fixed condition. **(B)** Head orientation RMS across target presentation phases by Δbehavioral-based trials (positive vs. negative). The mean RMS of yaw head orientation across eight trials in each phase (initial, middle, and final) for T0M0(SRT), T0M30(SRT), T0M30(%), and FSB(°). **(C)** The mean head orientation variability in the four conditions across the presentation phases by Δbehavioral-based trials. The mean SD of yaw head orientation across eight trials in each phase for test conditions. The error bars represent the standard error, and statistical significance is indicated by one asterisk for *p* < 0.05 and two asterisks for *p* < 0.01.

Linear mixed models (LMMs) were applied to all conditions, with Δbehavioral-based trial classification as a group factor and head condition as a within-subjects factor. A significant interaction effect (trials × head condition) was observed across all conditions: T0M0(SRT) (*F*(1, 80) = 18.444, *p* < 0.001), T0M30(SRT) (*F*(1, 80) = 16.377, *p* < 0.001), T0M30(%) (*F*(1, 80) = 12.974, *p* < 0.001), and FSB(°) (*F*(1, 80) = 14.229, *p* < 0.001). Based on the Δbehavioral-based classification, performance in negative trials significantly worsened (all *p* < 0.01), while performance in positive trials significantly improved when participants were free to move their heads (all *p* < 0.01, except for FSB in positive trials: *p* < 0.05; Figure 5A for details). Regarding the comparison of behavioral hearing outcomes between positive and negative trials under both head-fixed and head-free conditions, no significant difference was found in the head-fixed condition across test conditions (all *p* > 0.05), except for T0M0(SRT) (*p* = 0.002). In contrast, in the head-free condition, significant differences were observed in all conditions (all *p* < 0.01).

### 3.8 Δbehavioral-based trials comparison II: head orientation RMS and variability across target presentation phases

As shown in Figure 5B, the mean yaw orientation RMS was compared across trials categorized as “positive” and “negative” subsets, based on Δbehavioral, across the three target presentation phases (initial, middle, and final). In T0M0(SRT), a significant main effect of Δbehavioral-based trials was observed, with a 7.0° difference (*F*(1, 40) = 5.411, *p* = 0.025). The main effect of presentation phase was not significant, but the interaction between them approached significance (*F*(2, 40) = 3.101, *p* = 0.056). The analysis revealed that only the Δbehavioral-based trial effect reached significance in both T0M30(SRT), with an 8.5° difference (*F*(1, 40) = 4.586, *p* = 0.038), and FSB, with a 7.7° difference (*F*(1, 40) = 7.214, *p* = 0.010). No significant effect was observed in T0M30(%). As a result, positive trials yielded greater head orientation angles than negative trials across all conditions, except for T0M30(%), with a difference of approximately 7–8°.

For yaw orientation variability (Figure 5C), no main effects were observed in T0M0(SRT) and T0M30(%). However, in T0M30(SRT), significant main effects of Δbehavioral-based trials (*F*(1, 40) = 5.856, *p* = 0.020) and presentation phase (*F*(2, 40) = 3.567, *p* = 0.038), as well as their interaction (*F*(2, 40) = 3.423, *p* = 0.042), were detected. *Post-hoc* analysis revealed significant differences of 9.8° and 8.1° in yaw orientation variability between the two trials during the middle and the final phases (both *p* < 0.05), but not during the initial phase. Furthermore, yaw orientation variability for the positive trials increased significantly by 8.9° and 7.2° from the initial phase to the middle phase (*p* = 0.026), and then to the last phase (*p* = 0.029), whereas no significant changes were observed in the negative group. In FSB(°), a significant Δbehavioral-based trials effect was found, with a 5.7° difference (*F*(1, 40) = 4.632, *p* = 0.037).

### 3.9 Learning and fatigue effect

No significant main effect of repetition was observed in head-fixed SRTs for all conditions except T0M30(SRT) (*F*(2, 26) = 7.741, *p* = 0.002) where SRT significantly increased (i.e., worsened) by 4.4 dB TMR through the first session to the third session (*p* = 0.008). With regard to head-free results, no significant repetition effect was observed for all conditions except FSB (*F*(2, 26) = 4.124, *p* = 0.028) where spatial boundaries significantly worsened by 10.5° from the first to the second session (*p* = 0.027). In addition to the behavioral hearing outcomes, no repetition effect was observed in head orientation RMS and SD across yaw, roll, and pitch.

## 4 Discussion

### 4.1 Behavioral hearing outcomes between head-fixed and head-free condition

The lack of a significant difference in SRTs between head-fixed and head-free conditions when the target and masker were co-located was neither expected nor surprising, as head turns would induce identical spatial cue changes for both the target and maskers, preserving their relative configuration. This aligns with previous findings (Grange and Culling, 2016b). On the other hand, no significant difference in behavioral hearing outcomes was observed between conditions with spatially separated maskers, suggesting that natural head orientation was not advantageous to the listener. Although the direct comparison showed no significant difference, further analysis of the mixed effects of head rotation on speech performance uncovered a distinct bidirectional pattern in the Δbehavioral-based trial comparison. Negative Δbehavioral was associated with smaller head orientations and lower variability, implying that slight head turns impaired performance relative to the head-fixed condition, whereas positive Δbehavioral was linked to larger head rotations and movements, as shown in Figure 5.

Although not necessarily optimal, larger head rotations could produce greater contrasts in spatial cue changes between the target and the spatially separated masker streams, potentially providing updated favorable static or dynamic cues for segregation as compared to the head fixed conditions. Genzel et al. (2018), for example, showed dynamic cues generated by translational self-motion enable the auditory system to perceive relative depth (distance differences) between sound sources. Moreover, although the dynamic cue did not originate from listeners' head motion but rather from the movement of the target source itself, Cho and Kidd (2022) demonstrated that auditory motion in azimuth can serve as an effective time-varying spatial cue for segregating a target source among competing talkers, even in the absence of static spatial separation cues. The magnitude of target motion was positively associated with the degree of this effect, which aligns with the positive Δbehavioral observed in our participants exhibiting greater head rotation magnitudes and variability. This finding suggests that listeners could utilize dynamic spatial cues arising from head movements to achieve better segregation than under the head-fixed condition.

On the other hand, previous studies reported unfavorable effects of changes in head orientation, noting that participants under natural head-orientation conditions appeared to move their heads insufficiently or less optimally compared to when head turns were instructed (Grange and Culling, 2016a; Grange et al., 2018). Hendrikse et al. (2018) also found no evidence indicating that subjects actively used head movements to optimize the signal-to-noise ratio or to adopt a localization-driven head movement strategy. Indeed, when listeners were allowed to move their heads in this experiment, they exhibited high inter-subject and intra-subject variability in head orientation and movement rather than maintaining an optimal angle. However, with respect to head-orientation RMS, although the difference was statistically significant, the mean RMS for negative trials was not substantially different from that of positive trials or the head-fixed condition, making it difficult to interpret these orientations as undesirable, particularly under the limited better-ear hearing condition. Nevertheless, inadequate head orientation and movement may result in dynamic cues that are insufficient to facilitate better segregation. Rather, slight, transient, and infrequent head turns appeared to disrupt the ongoing auditory streaming, leading to an undesirable reorganization (i.e., reset) that adversely affected speech-on-speech intelligibility (Kondo et al., 2012).

### 4.2 Head behavior and active listening

Importantly, direct comparisons between the head-fixed and head-free conditions did not reveal significant performance differences across conditions. However, inspection of the individual data (Figures 2B, F, 3B, F) revealed heterogeneous outcomes, with some listeners showing performance improvement in the head-free condition (positive benefit) and others showing deterioration (negative benefit). Because head movements in this study were natural and undirected, participants were free to move their heads in different ways across trials, resulting in a wide range of head-movement magnitudes. Previous studies have also suggested that the relationship between head movement and performance is not necessarily monotonic. Therefore, a *post-hoc* grouping approach was used in an exploratory analysis to examine whether the bidirectional performance changes were associated with distinct head-movement patterns, such as head-orientation magnitude and movement variability.

The bidirectional pattern observed in the Δbehavioral-based trial comparison cannot be fully explained solely by listeners' utilization of dynamic spatial cues. Rather, it appears to be associated with differences in head-orientation behavior. Head orientation variability indicates how dynamically participants moved their heads across trials; positive trials exhibited greater head movements than negative trials. Considering the characteristics of the adaptive test procedure, head movements in positive trials varied with task difficulty across phases, whereas negative trials were characterized by both smaller head rotation and a lack of such phase-dependent changes. This pattern may reflect how actively participants engaged with the spatial hearing task, with positive trials representing more active listening and negative trials reflecting more passive listening.

In this study, head movement can perhaps be interpreted as a spontaneous behavior associated with active listening (i.e., task engagement), rather than as an intentional strategy for optimizing spatial hearing. Indeed, changes in performance due to natural head behaviors (Δbehavioral) were not consistent across the four listening configurations, as indicated by the lack of significant correlations in Δbehavioral across these conditions (see Table 1). Valzolgher et al. (2024a) similarly suggested that head movement is not merely a strategy for achieving functional gains in spatial hearing, but is also associated with reduced subjective listening effort and increased confidence. In parallel, in visual processing tasks, head movement may not simply reflect a passive response but rather serve as an active strategy to improve task efficiency and reduce cognitive load (Risko et al., 2014). Larger or more frequent head movements have been reported in more complex listening environments. For example, increasing acoustic scene complexity, manipulated through the number of talkers and reverberation, leads to more frequent and more variable head-movement behavior during auditory scene exploration (Slomianka et al., 2024). Moreover, greater head movement was observed in the high informational masking condition than in the low informational masking condition (Brimijoin et al., 2012). Accordingly, it can be inferred that the negative Δbehavioral observed in this study might result from passive listening with lower engagement.

### 4.3 Natural head orientation tendencies

Consistent with previous research (Thurlow et al., 1967), listeners in the present study exhibited greater yaw head orientation than pitch or roll orientation in spatial hearing along the horizontal plane, as indicated by significantly larger yaw RMS values. Under head-free conditions, the average head orientation RMS was in the range of 15° to 21°. These modest head orientations could be explained by the location of target and maskers. Listeners may not have perceived head movement as necessary for listening to the target coming from the front (Tetard et al., 2023). Hendrikse et al. (2018) observed that most participants remain stationary and consistently direct their gaze toward the center in audio-only conditions without visual cues. Another possibility is that listeners tended to move their heads within the range of the separated masker locations (±30°) or within the FSB(°) to avoid degradation of the inter-source contrast in spatial cues.

Despite a significant main effect of listening condition on head orientation, pairwise comparisons did not reveal any significant differences in head orientation RMS. This may be attributed to the small angular separation between target and maskers as well as high variability across participants (Figure 4). Given the skewness of the raw head orientation data distribution (within ±0.12; see panels C and G in Figures 2, 3), and the similarity between the mean and median near zero, listeners appeared to show no lateral orientation bias in either the co-located or the symmetrically separated masker conditions. In addition, participants did not display a systematic pattern of orienting toward an optimal angle across the three repeated trials, as there was no significant change in head orientation RMS or SD, nor was there any learning effect on behavioral hearing outcomes from the first to the third trial. This tendency might reflect the limited benefit of better-ear listening in this study.

### 4.4 Limitation

The current experimental setting for the spatial-hearing measures may not have provided listeners with a sufficiently sensitive listening configuration to utilize dynamic spatial cues and their inter-source contrasts for several reasons. First, the fixed angular separation of 30° between the target and symmetric maskers was modest, as the maskers were positioned close to the target. This configuration could have restricted the potential range of head orientations, resulting in considerable overlap in azimuth relative to the facing-forward position. Second, the use of a different-gender masker may have led to dominance of spectral cues, thereby reducing the reliance on spatial cues for segregation (Oh et al., 2023). This could be attributed to the number of maskers (i.e., two speech maskers plus four multilingual conversations). Yost (2017) reported that SRM was nearly eliminated when six masker sources were used, likely due to the difficulty of accurately localizing more than five simultaneously presented speech sources, which in turn impairs selective attention. This likely indicates that listeners already have trouble with binaural processing (binaural unmasking) and auditory selective attention, irrespective of head movement. This phenomenon appears to be consistent with no relationship between head movement and speech discrimination in a diffuse noisy condition (Gifford et al., 2018; Tetard et al., 2023). Grange and Culling (2016b) also reported a significant but slight improvement of only around 1 dB in a virtual environment with multiple interfering sound sources. Third, the experimental paradigms described here did not provide performance feedback to participants. It is possible that feedback would motivate participants to explore additional means such as head movement to boost performance. Lastly, because intermediate azimuths in the FSB task were generated using equal-power panning, the resulting phantom sources were not acoustically identical to physical loudspeaker sources, although amplitude panning has been used in spatial hearing research, including studies of spatial release from masking (Pastore and Yost, 2017, 2022).

## 5 Conclusions

Allowing natural, undirected head movement did not produce group-level improvements in behavioral hearing outcomes for either co-located or modestly separated symmetric maskers. However, correlation analyses indicated that larger head orientations were associated with more positive performance changes relative to the head-fixed condition in the co-located SRT and FSB conditions. Exploratory analyses further suggested a bidirectional pattern in trial-wise Δbehavioral across conditions in which either masker level or location was adaptively varied, but not in the condition where both were fixed: smaller and less variable head turns were associated with poorer outcomes relative to the head-fixed condition, whereas larger and more variable movements accompanied performance improvements. These patterns may reflect interactions between dynamic spatial cues produced by head motion and moment-to-moment variations in listener engagement or listening behavior.

Taken together, the present findings suggest that natural head movement may not function as a uniformly beneficial or deliberate listening strategy, but rather as a listener-dependent behavioral correlate of performance during spatial listening tasks. Further research is needed to clarify how spontaneous head movements interact with spatial cues and task engagement in complex auditory environments.

## Data Availability

The datasets generated in this study are publicly available on the Open Science Framework (OSF) at https://osf.io/ch3as.
